# miRNA-mRNA Integrated Analysis Reveals Roles for miRNAs in Primary Breast Tumors

**DOI:** 10.1371/journal.pone.0016915

**Published:** 2011-02-22

**Authors:** Espen Enerly, Israel Steinfeld, Kristine Kleivi, Suvi-Katri Leivonen, Miriam R. Aure, Hege G. Russnes, Jo Anders Rønneberg, Hilde Johnsen, Roy Navon, Einar Rødland, Rami Mäkelä, Bjørn Naume, Merja Perälä, Olli Kallioniemi, Vessela N. Kristensen, Zohar Yakhini, Anne-Lise Børresen-Dale

**Affiliations:** 1 Division of Surgery and Cancer, Department of Genetics, Institute for Cancer Research, Oslo University Hospital Radiumhospitalet, Oslo, Norway; 2 Department of Computer Science, Technion, Haifa, Israel; 3 Medical Biotechnology, VTT Technical Research Centre of Finland, Turku, Finland; 4 Institute of Clinical Medicine, Faculty of Medicine, University of Oslo, Oslo, Norway; 5 Agilent Laboratories, Tel Aviv, Israel; 6 Department of Informatics, University of Oslo, Oslo, Norway; 7 Division of Surgery and Cancer, Department of Oncology, Oslo University Hospital, Oslo, Norway; 8 Institute for Molecular Medicine Finland, University of Helsinki, Helsinki, Finland; 9 Institute for Clinical Epidemiology and Molecular Biology (EPIGEN), Nordbyhagen, Norway; 10 Division of Pathology, Oslo University Hospital, Oslo, Norway; Baylor College of Medicine, United States of America

## Abstract

**Introduction:**

Few studies have performed expression profiling of both miRNA and mRNA from the same primary breast carcinomas. In this study we present and analyze data derived from expression profiling of 799 miRNAs in 101 primary human breast tumors, along with genome-wide mRNA profiles and extensive clinical information.

**Methods:**

We investigate the relationship between these molecular components, in terms of their correlation with each other and with clinical characteristics. We use a systems biology approach to examine the correlative relationship between miRNA and mRNAs using statistical enrichment methods.

**Results:**

We identify statistical significant differential expression of miRNAs between molecular intrinsic subtypes, and between samples with different levels of proliferation. Specifically, we point to miRNAs significantly associated with TP53 and ER status. We also show that several cellular processes, such as proliferation, cell adhesion and immune response, are strongly associated with certain miRNAs. We validate the role of miRNAs in regulating proliferation using high-throughput lysate-microarrays on cell lines and point to potential drivers of this process.

**Conclusion:**

This study provides a comprehensive dataset as well as methods and system-level results that jointly form a basis for further work on understanding the role of miRNA in primary breast cancer.

## Introduction

Expression profiling of mRNA has been used to molecularly characterize various tissues and tumors. A range of gene signatures that predicts pathway activation, has been identified in various cancer types (reviewed in [1]). In breast cancer, mRNA profiling has been used to classify breast tumors and associate them with clinical and pathological characteristics as well as with prediction of outcome [2], [3], [4], [5]. In particular, luminal-A and basal-like subtypes, defined using an intrinsic gene list, have distinct and reciprocal gene expression profiles as well as large differences in clinical characteristics, including survival [5], [6], [7], [8].

Gene expression regulation through mechanisms that involve microRNAs (miRNAs) has attracted much attention during recent years. miRNAs are a class of endogenous small regulatory RNA molecules that target mRNAs and trigger either translation repression or mRNA degradation [9]. There are to date more than 900 identified human miRNAs [10], transcribed as individual units, polycistronic clusters or in concert with a protein coding host gene [11]. Many miRNAs regulate genes associated with different biological processes such as development, proliferation, apoptosis, stress response, and tumourigenesis [12], [13], [14], [15], [16].

Abnormal expression levels of several miRNAs have previously been shown to be associated with multiple cancer types including breast cancer [17], [18], [19], [20]. Some miRNAs correlate with specific clinical features of breast cancer, such as estrogen and progesterone receptor expression, tumor stage, vascular invasion, and proliferation index [19], [21], [22], [23]. In a study by Blenkiron et al. a set of 309 miRNAs were profiled in 93 human primary breast tumors, 5 normal breast samples and 21 cell lines, identifying 31 miRNAs associated with molecular subtype, estrogen receptor status or grade [24]. In addition, this study reported a strong co-regulation of miRNA genomic clusters and showed that for the majority of miRNAs differential expression cannot be attributed to chromosomal loss or gain in their genomic region. The study reports some findings that pertain to jointly analyzing the miRNA data with its matching mRNA data. Continuing this direction and taking a systematic approach to joint analysis will further enhance our understanding of the role of miRNA in breast cancer pathogenesis and progression.

In this work we present expression profiling of 799 miRNAs in 101 human primary breast tumor samples, along with genome-wide matched mRNA profiling and extensive clinical information. We applied several approaches to statistically analyze the resulting data. We identified statistically significant differential expression of miRNAs that distinguishes the reciprocal basal-like and luminal-A breast cancer subtypes. Our analysis confirmed some observations from previous studies including Blenkiron et al. [24], but also revealed subtype specific expression of previously uncharacterized miRNAs. We put emphasis on the joint analysis of miRNA and mRNA data, and analyzed correlations between miRNA and mRNA expression data. We show that particular cellular processes such as proliferation, cell adhesion, and immune response are significantly enriched in the co-regulated clusters, suggesting a central role for miRNAs in regulating these pivotal pathways. We performed functional assays using direct measurement techniques to validate the influence of miRNA on proliferation.

## Results

### miRNA differential expression in molecular breast cancer subtypes

**Figure 1:**
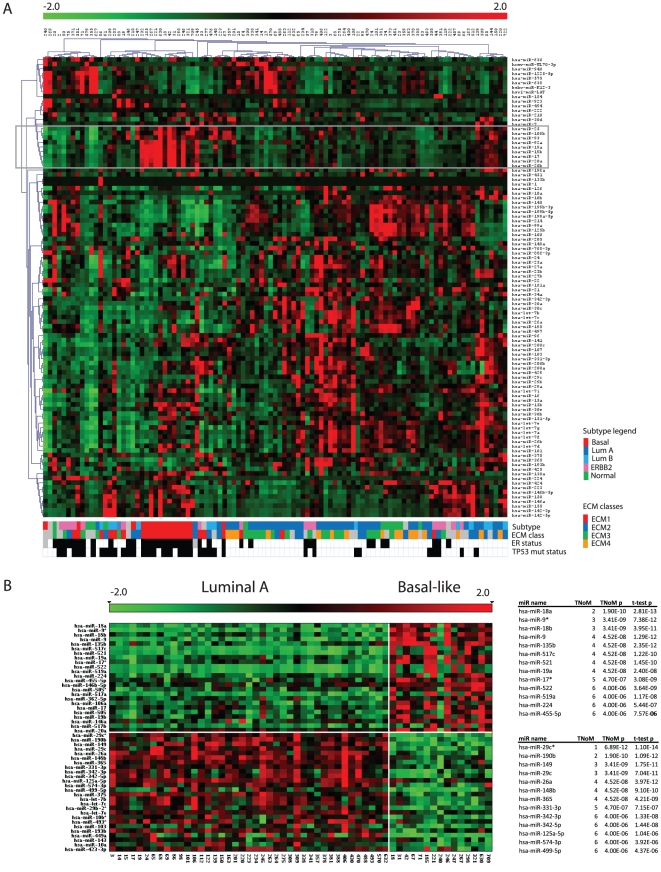
Subtype specific differential miRNA expression. (A) Hierarchical clustering of the 100 most variable (largest variance values) miRNAs across 101 tumors with dendrograms illustrating similarities between samples/genes. The yellow box indicates members of the miR-17-92 clusters that have higher expression in the basal-like subtype. The color in the bars beneath the heatmap illustrates the different subtypes as defined by centroid correlation [8] and EMT classes [29]. Black boxes represent ER- and *TP53* mutant samples. (B) miRNAs differentially expressed between basal-like and luminal-A subtypes. miRNAs ordered by significance of differential expression between 15 basal-like and 41 luminal-A samples. The 26 most significant of 77 miRNAs (p<0.0001) are shown in the tables with number of misclassified samples (TNoM), TNoM p-value, and t-test p-value. For visualization expression values of each miRNA were linearly stretched to a scale of -2.0 to 2.0.

miRNA expression profiling was carried out for 101 human primary breast cancer samples (Table S1) using microarrays covering 799 miRNAs, from Agilent Technologies. After filtering miRNAs that were not expressed in most of the cohort (see Materials and Methods), 489 miRNAs were considered for further analyses. Applying hierarchical clustering based on the 100 most variably expressed miRNAs, we observed a cluster consisting of tumors with mainly basal-like subtype characterization, that was distinguished by a higher expression of the miR-17-92 cluster/family from 7q22.1, 13q31.3 and Xq26.2 (Figure 1A).

Since the basal-like breast cancer subtype is defined by specific morphological and pathological characteristics as well as by a distinct mRNA expression profile we explored the full list of miRNAs related to the basal-like tumors. Comparing miRNA expression in 15 basal-like and 41 luminal-A samples, the subtypes with strongest reciprocal mRNA expression profiles, 111 differentially expressed miRNAs were identified at an FDR (False Discovery Rate [25]) of 5%. The top 26 miRNAs separated almost perfectly the basal-like and luminal-A samples (Threshold number of misclassification (TNoM) ≤6, see Materials and Methods). These results confirm that the subtype difference is manifested also at the level of miRNA expression (Figure 1B, Table S2). Only five miRNAs were intronic in genes that play a role in the mRNA subtype classification (hsa-mir-324 in *ACADVL*, hsa-mir-153 in *PTPRN2*, hsa-mir-934 in *VGLL1*, hsa-mir-595 in *PTPRN2*, hsa-mir-744 in *MAP2K4*), demonstrating that the miRNA differential expression is not merely a recapitulation of the mRNA classification.

The top miRNAs with elevated expression levels in basal-like samples were miR-18a/b (TNoM p<2E-10) and other members of the miR-17-92 cluster (miR-17/17*, miR-18a/b, miR-19a, miR-20a and miR-106a). In addition we found miR-9/9* (TNoM p-value < 4E-9), which had no detectable expression in most of the non-basal-like samples (Figure S1A). Among the prominently down regulated miRNAs in basal-like tumors were representatives of the miR-29 family (TNoM p<7E-12) along with miR-190b (TNoM p<2E-10) (Figure S1B-C). miR-29 family members were moderately expressed in subtypes other than luminal-A and basal-like. miR-190b had an almost discrete binary expression mode with higher expression in the luminal-A/B subtypes than in the basal-like/ERBB2-enriched subtypes (TNoM p<4E-15). We further discuss the role of miR-29 in our cohort in later sections.

### miRNA expression and TP53 mutational status

**Figure 2:**
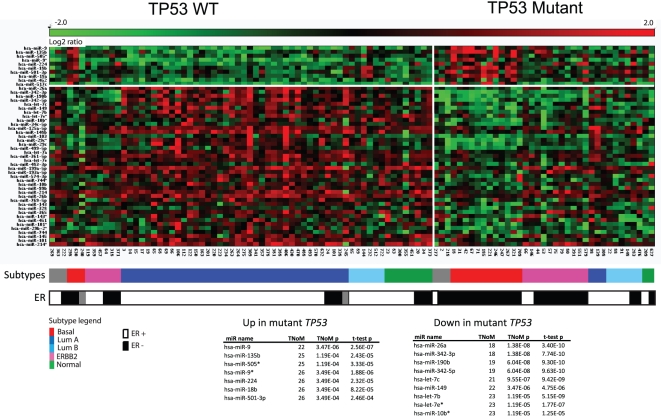
miRNAs differentially expressed between *TP53* WT and mutated samples. miRNAs ordered by significance of differential expression between *TP53* WT and mutant samples. Color boxes illustrate molecular subtype, and estrogen receptor status in black and white, while gray boxes represent unclassified samples. The 16 most significant of 44 miRNAs (p<0.0001) are shown in the tables with number of misclassified samples (TNoM), TNoM p-value, and t-test p-value. For visualization expression values of each miRNA were linearly stretched to a scale of -2.0 to 2.0.

Mutations in the *TP53* gene are well studied and have been associated with cancer progression and worse prognosis [26]. In the present cohort we explored the miRNAs that were differentially expressed between the 64 wild-type samples (WT) and 36 *TP53* mutant samples (Figure 2). Since the breast cancer subtype classification is not independent of the *TP53* status, with most of the basal-like and ERBB2-enriched tumors having *TP53* mutations, many of the same miRNAs were found to be differentially expressed in both partitions. At top among the 81 differentially expressed miRNAs (at 5% FDR) we identified miR-342-3p (TNoM p < 2E-08) to have significantly lower expression in the *TP53* mutant tumors. We note that miR-34a, previously shown to regulate TP53 [14], is not observed to be differentially expressed in comparing TP53 mutational status in this cohort (Table S3).

As previously reported [26] there is a strong association between estrogen receptor (ER) and *TP53* status (Figure 2). Tumor samples with wild type *TP53* are mostly ER-positive and samples with mutated *TP53* are mostly ER-negative. In line with this co-occurrence we observed a substantial overlap between miRNAs differentially expressed between ER+ and ER- samples and between WT and mutant *TP53* samples. Removing this confounding factor, by comparing 50 *TP53* WT/ER+ samples vs. 11 *TP53* mutant/ER+ samples or 12 *TP53* WT/ER- samples vs. 26 *TP53* mutant/ER- samples, we observed different repertoires of differentially expressed miRNAs (Table S3).

### Joint enrichment analysis associates miRNAs to distinct biological processes

To better understand the role of miRNAs in different biological modules, as evidenced in our cohort of primary breast cancer samples, we further take a systems biology approach to examine the correlative relationship between miRNA and mRNAs. For each miRNA, separately taken as a pivot, the mRNA transcripts were ranked according to the correlation of their expression pattern to the expression pattern of the pivot miRNA. Using GO enrichment analysis carried out on the ranked list of mRNAs (see Materials and Methods) we were able to elucidate the biological modules that are correlated or anti-correlated to the expression level of the pivot miRNA (Table S4). We note that this association does not imply a direct regulation by the miRNA but rather indicates the biological process in which the pivot miRNA plays a role. For several cases we are able to find an enrichment of the pivot miRNA targets in its anti-correlated mRNAs. In these cases we point to a potential direct regulation effect by the miRNA.

Several pivot miRNAs showed strong association to cell-cycle genes. Specifically, we observed an enrichment of cell-cycle genes when considering positive correlation to members of the miR-17-92 cluster, with miR-93 and miR-18b yielding the strongest enrichment (minimum-hypergeometric (mHG) p<E-74 and p<E-73, respectively). In accordance with previous results [27] we also observed a significant enrichment of genes regulated by E2F (mHG p<E-24 and p<E-27 for the above two miRNAs, respectively; see Materials and Methods). We note that miR-19b targets are enriched amongst its anti-correlatees (mHG p<2E-12). For miR-493 and the miR-214 cluster we observed cell-cycle genes to be enriched in the negatively correlated genes.

**Figure 3:**
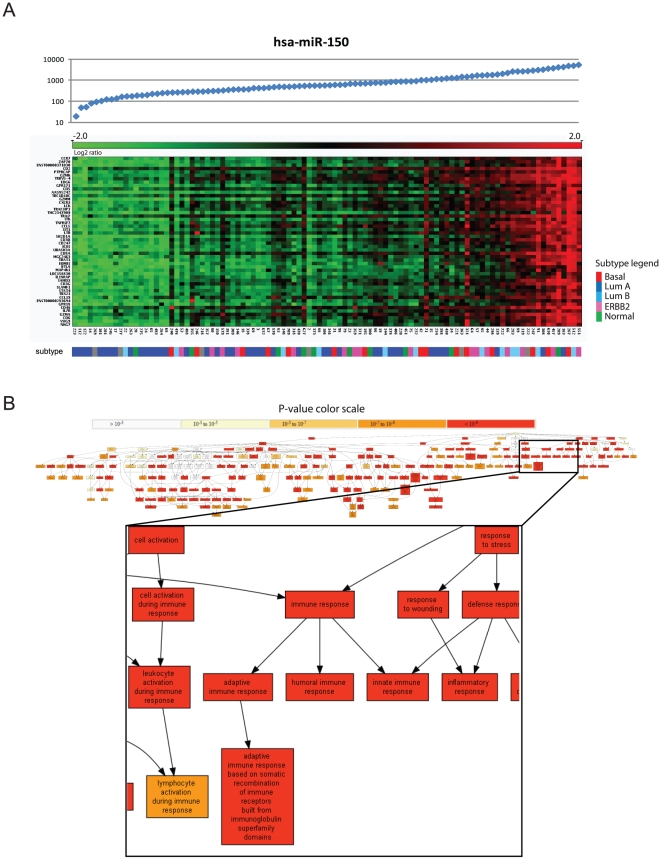
Expression of miR-150 and its mRNA correlates. (A) The samples are ordered according to the expression levels of miR-150 for that sample. The absolute signal intensities of miR-150 are presented in the top panel. The top 50 correlated genes are sorted from top to bottom. The color in the bar beneath the heatmap indicates the different subtypes. As can be seen many luminal-A samples have low levels of miR-150 expression though no clear cut can be deduced to separate the luminal-A samples from the rest of the subtype samples. (B) Graphical representation of GO-term enrichment of genes positively correlated to miR-150. The strongest enrichment is seen for the “immune response” (p<1.2E-147) term. The graph is color coded according to degree of enrichment. Figure obtained using the GOrilla web tool [62].

Another biological module with many significantly associated miRNAs is that of the immune response (Table S4). In particular, miR-150 was found to have the strongest enrichment of the immune response term amongst its positive correlated mRNAs (mHG p<E-147, Figure 3). In addition we identified other miRNAs with a strong positive correlation to the immune response module (e.g. miR-146 at mHG p<E-132, miR-142 at mHG p<E-108, miR-155 at mHG p<E-123, and miR-223 with mHG p<E-111). Interestingly, their association was more related to T-cell activation genes while miR-150 was strongly associated with genes related to the inflammatory response.

### miRNA expression associated with proliferation

**Figure 4:**
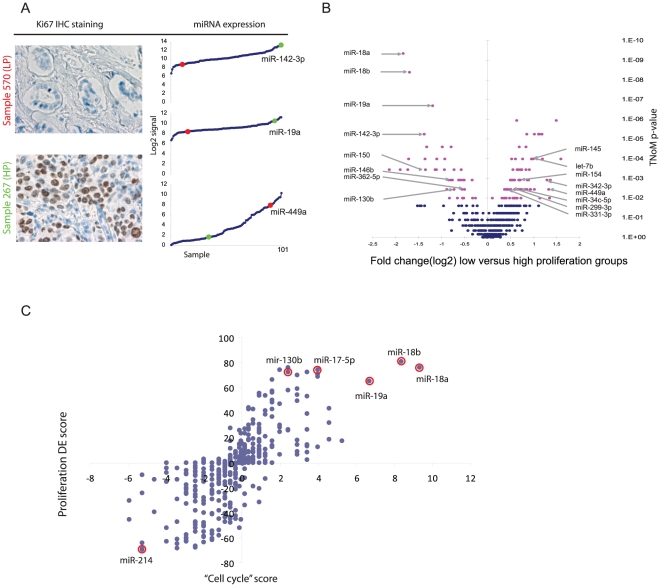
Proliferation associated miRNAs. The panels show miRNAs that are both positively and negatively associated with proliferation in *in vivo* profiling from tumors. (A) Immunohistochemistry staining of Ki67 of tumors scored as highly proliferative (HP, sample 267) and low proliferative (LP, sample 570). The right panel shows the signal distribution in the 101 samples for three selected miRNAs, miR-142-3p, miR-19a, and 449a, with signal intensities for sample 570 (red dot) and 627 (green dot) highlighted. (B) Volcano plot of all miRNAs with TNoM p-value of differential expression against fold change differences in low (24 samples) versus high (35 samples) proliferation groups. Pink dots represent significant miRNAs (p<0.001). Left part contains miRNAs that are up-regulated in highly proliferative samples and right part down-regulated miRNAs. (C) The plot shows the scores for each miRNA, where each miRNA is represented by a dot. On the Y-axis differential expression score is –log(p-value) if miRNA is up-regulated and log(p-value) if miRNA is down regulated, thus assigning positive and negative scores according to differential expression between the high and low proliferative groups. Significance of differential expression is calculated using TNoM as described in Materials and Methods. “Cell-Cycle” (CC) score is –log(p-value) if the CC genes are enriched within the genes positively correlated to the miRNA, and log(p-value) if the CC genes are enriched within the genes negatively correlated to the miRNA. p-value for CC enrichment is calculated using the mHG statistic as described in Materials and Methods.

Our systematic approach led us to pay special attention to the cell-cycle module in the context of our cohort of early stage breast cancer patients. As activation of cell-cycle genes is closely related to proliferation we further examined the expression of miRNAs in samples with different proliferative states. To do so, we used Ki67 immunohistochemistry staining (IHC) and scoring of mitotic count from tumor sections to partition the samples into a High-Proliferative class (HP), consisting of 24 samples, and a Low-Proliferative class (LP) consisting of 35 samples (Figure 4A, see Materials and Methods). We identified 123 differentially expressed miRNAs (at 5% FDR, Table S5) when comparing HP and LP classes. This high number reflects the significant difference between the miRNA expression signatures of the two classes (Figure 4B, Table S5).

No basal-like nor luminal-B samples were assigned to the LP class, while no luminal-A and no normal-like samples, except one, were grouped to the HP class. However, ERBB2-enriched samples were equally assigned to the different proliferation classes (6 in HP and 5 in LP). To test for subtype independent miRNA differential expression we examined differential expression using only the ERBB2-enriched samples. We identified 21 miRNAs (at TNoM p<0.05) that were associated with proliferation, of which 7 were also among the differentially expressed miRNAs in the overall comparison (Table S5). Specifically, miR-574-3p and miR-18b ranked high in both comparisons.

Estrogen receptor (ER) status differs between the LP (6 ER-; 29 ER+) and HP (17 ER-; 7 ER+) groups. We further examined the association of miRNAs to proliferation in the ER-negative samples. While most miRNAs (e.g. miR-18b up-regulation and miR-145 down-regulation) showed an ER status independent differential expression, miR-199 and miR-214 were down-regulated in proliferating samples only in the 38 ER-negative samples (both with TNoM p<0.01, see Table S6).

To better understand the relation of the miRNAs to different proliferating processes we compared the cell-cycle associations as described above to the level of differential expression, according to proliferation status (HP vs. LP), of the miRNAs. As would be expected we find the miRNAs associated with cell-cycle genes to be differentially expressed between proliferative samples and non-proliferative samples. However, we also observe that the level of differential expression of the miRNAs in proliferation is in monotone relation to the level of enrichment of the cell-cycle genes (Figure 4C). Moreover - there is a strong correlation between the extent of miRNA over-expression in proliferative samples and the enrichment of the GO term “positive regulation of mitotic cell cycle” in its correlated genes (Pearson's r  = 0.76, Figure S2A). An opposite observation holds for the GO term “negative regulation of S phase of mitotic cell cycle”, where miRNAs over-expressed in the proliferative samples show enrichment of this GO term in their anti-correlated genes (Pearson's r  =  −0.51, Figure S2B). We further analyzed all GO terms to explore the relationship between enrichment and HP vs. LP differential expression. The strong positive correlation observed in Figure 4C for the cell-cycle module is not attained by any other GO term.

### Functional characterization of proliferative miRNAs

**Figure 5:**
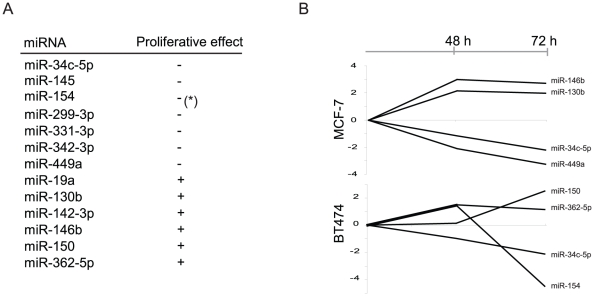
Proliferation assay in miRNA transfected cell-lines. The panels show miRNAs that are both positively and negatively associated with proliferation in transfected cell-lines. (A) list of miRNAs that showed a significant effect on proliferation in cell-lines with a corresponding differential expression in tumors. (* indicates opposite effect in MCF-7 and BT-474). (B) Lysate microarray (LMA) screening of MCF-7 and BT-474 cells transfected with 20 nM human Pre-miR™ miRNA Precursor library v2. Ki67 readout after 48 and 72 hours. The two miRNAs from panel A with strongest positive and negative effect on proliferation for each cell line are shown (see Table S7 for complete list)

In order to indentify miRNAs that potentially drive the proliferation process in breast cancer we tested a subset of the HP vs. LP differentially miRNAs using high-throughput lysate microarray (LMA) screening technology [28]. For that purpose, miRNAs were transfected into the cell lines MCF-7 (luminal-like, ER-positive) and BT-474 (ERBB2-amplified, ER-positive, TP53 mutated) using a library of pre-miR constructs. After 48 h and 72 h incubation, the cells were lysed and the lysates were printed onto nitrocellulose-coated slides and stained with a specific antibody against Ki67 to assay the effect of miRNA on proliferation (see Materials and Methods). Of the 123 miRNAs identified as LP vs. HP differentially expressed, 61 were represented in the LMA/Ki67 screen. Of these 13 showed a matching effect on Ki67 protein level in the tested cell lines (Figure 5A, Table S7). Among the miRNAs that were down-regulated in HP samples we found the strongest matching effect for miR-449a (MCF-7), miR-154 (BT-474) and miR-34c-5p (MCF-7 and BT-474) (Figure 5A and B). In the MCF-7 cell line over-expression of miR-342 leads to reduced proliferation, which is in line with its low expression in TP53 mutated samples. Of those over-expressed in the HP samples we found the strongest matching effect for miR-146b (MCF-7) and miR-150 (BT-474), but we also see an agreement for miR-19a and miR-130b (Figure 5A and B and Table S7). Among the miRNAs with opposite expected effect on Ki67 levels we note miR-18a/b and let-7b/c/e (See Table S7 for complete list).

### miR-29c is associated with cell adhesion/extra cellular matrix

The tumors studied here have previously been classified into four classes based on a signature of extracellular matrix (ECM) genes [29]. Strong degree of differential expression is seen at the miRNA level between ECM1 and ECM3 as well as between ECM1 and ECM4. We focused on the ECM1 and ECM4 classes (23 and 16 samples, respectively) and identified 47 differentially expressed miRNAs (at 5% FDR, Figure S3A). miR-29c, shown in previous sections to be down regulated in basal-like samples as compared to luminal-A samples, was found to be the most differentially expressed miRNA between the two ECM classes (TNoM p<3E-5, lower in ECM1, Table S8). We used the TargetScan prediction tool [30] to rank all genes according to their miR-29c target prediction scores. The top predicted targets of miR-29c were found to be enriched with genes related to the cell-adhesion GO-term (Figure S4). Further supporting its direct role in our cohort, we found miR-29c targets as well as cell adhesion genes to be significantly anti-correlated with miR-29c expression (mHG p<2E-11 and mHG p<2E-13, respectively; see Figure S3B and C and Materials and Methods).

### miRNAs related to survival

For all 489 miRNAs, we ran univariate Cox regressions to predict survival. The top 9 miRNAs resulted in 37% FDR (Table S9).

High expression levels of the immune module have previously been associated with better survival in ER-/HER2- patients [31]. In agreement with the strong association of miR-150 with genes related to the immune response, we found high expression of miR-150 alone to be predictive of better prognosis (log-rank p<0.085) within the corresponding set of patients in our cohort, namely the ER-/non-ERBB2 enriched patients (Figure S5).

## Discussion

In this work we introduce an extensive analysis of miRNA expression in 101 tumor samples from breast cancer patients. We show that miRNA expression alone is sufficient to distinguish luminal-A from basal-like samples, two types that represent different degrees of aggressiveness of the disease. Specifically, the oncogenic miR-17-92 module is shown to be distinctly over-expressed in highly proliferative samples including the basal-like samples, which are also characterized with high frequency of *TP53* mutations. Recently we have shown that the miR-17-92 module is repressed by wild-type *TP53* in an E2F1-mediated manner [27]. Our integrated analysis supports the E2F association with the miRNA cluster. Other over-expressed members of the cluster, such as miR-17-5p/miR-20, have been linked to the regulation of cell proliferation through a Cyclin D1 regulatory feedback loop [32] and through the inhibition of AIB1 translation [33] in breast cancer. miR-18a directly targets ESR1 [28] and has been shown to promote estrogen receptor alpha (*ESR1*) dependent proliferation in hepatocellular carcinoma cells [34]. In addition we have recently observed that the miR-18 cluster is over-expressed in a panel of samples from various cancers types [20]. miR-18a/b may therefore be an important contributor to the different overall gene expression profiles that distinguish between malignant to non malignant tumors, specifically ER positive tumors.

We further explore and characterize the miRNA expression signature that distinguishes between basal-like and luminal-A samples, known to be reciprocal with respect to mRNA expression and clinical properties. We show that miR-9/9* are markers for aggressive tumors, being expressed specifically in the basal-like tumors. miR-9/9* are over-expressed in c-Myc induced mouse mammary tumors [35], as well as in brain [36] and ovarian primary cancers [37] which points to a more general role in cancer progression for this miRNA. In our cohort we see a positive correlation between miR-9 and c-Myc (Pearson's r = 0.22) and further work is needed to better understand the interplay between the two factors in breast cancer.

In addition to tumor subtypes we also analyzed the miRNA expression signature with respect to other molecular characteristics of the tumors like *TP53* mutations. *TP53* mutant samples largely overlap the basal-like samples and the ER-negative samples. Therefore the majority of the miRNAs found to be over-expressed in basal-like samples were also over-expressed in *TP53* mutated samples and ER-negative samples and vice versa. Among the differentially expressed miRNAs we find miR-34c which is a direct transactivation target of *TP53* [38] and miR-18a/b which targets ER [28]. The let-7 family and miR-342 exhibit, in our cohort, a more significant differential expression between TP53 mutational statuses than between ER statuses or tumor subtypes. These miRNAs have previously been linked to tumorigenesis [39], [40] and further characterization is needed to understand their relationship to *TP53* and ER. Although miR-34a was previously shown to regulate TP53, we do not see it differentially expressed in the context of TP53 mutational status which might indicate a lack of feedback loop in this regulation.

We introduce several new approaches to analyze miRNA and mRNA expression data in an integrated manner using a systems biology approach. In particular we assess the enrichment of various gene sets amongst the genes correlated/anti-correlated to the expression levels of a pivot miRNA. Our straight forward approach allows to comprehensively associate miRNAs to biological processes in a statistically sound and functionally relevant manner. Thus, our approach leads us to additional insight into the role of miRNAs in breast cancer and enables the identification of key players. Other approaches of data integration [41], [42] are aimed at finding a set of miRNAs and a set of mRNAs that are expressed in a concerted manner either in a subset of the samples or in the entire cohort. We note that while these approaches are computationally sophisticated they are not driven by functional relevance.

In particular we point to a significant association of many miRNAs to the cell-cycle module. Our methodology also shows that miRNAs over-expressed in proliferative samples, are correlated to positive regulators of cell-cycle. Similarly, miRNAs under-expressed in proliferative samples are correlated with negative regulators of cell-cycle. The relationship between enrichment of these gene sets and proliferation related differential expression is, in general, monotone, as depicted in Figure 4. This monotonicity further demonstrates the sensitivity of our approach in terms of detecting miRNA association to biological processes.

The integrated analysis also revealed the association of several miRNAs to the immune response module, a major biological process closely associated with cancer progression and development [43]. We found miR-150, as well as miR-155 and miR-142, to have strong positive correlation to the immune response module. As miRNAs are considered to be negative regulators of expression the positive association of miR-150 to the immune response indicates that miR-150 is not a direct regulator of this process but rather a part of the immune response transcriptional program. Several studies have identified miR-150 to be involved in controlling B-cell differentiation by targeting the transcription factor c-Myb [44], [45], [46]. In our dataset (data not shown) we see an inverse correlation (Pearson's r  =  −0.18) between Myb and miR-150 expression levels which might point to a similar regulation pathway of immune response in breast cancer. Immune response was previously linked to clinical outcome in ER-/HER2- samples [31], and in accordance with its strong association to the immune response module we see that miR-150 can predict clinical outcome in our ER-/HER2- samples. This makes miR-150 a good marker for the activity of the immune response in breast cancer samples, as high expression of miR-150 is associated with active immune response and better prognosis. Since we could not find any association of miR-150 to any of the known breast cancer subtypes we propose that its expression can act as a mean for classifying breast cancer samples based on immune response. However, more studies are needed to better elucidate causal relationships, if any, between miR-150 and the immune response as well as between miR-150 and prognosis.

We further examined the involvement of miRNAs in regulating cell proliferation. Comparing the highly proliferative (HP) samples to the low proliferative ones (LP) we discovered a distinct miRNA expression signature. Specifically, we validate in vivo the association of the miR-17-92 module to proliferation in breast cancer, in agreement with the same association observed in tumor cell lines [47]. In addition, we find miR-199a and miR-214 to be significantly down regulated in HP samples, specifically in ER-negative samples. Both miRNAs reside on 1q24.3 and were previously shown to be downregulated in ER- samples [24] and to induce cell survival by targeting PTEN and subsequently activating the Akt pathway in ovarian cancer [48]. Overall, we observed a higher number of miRNAs with lower expression in HP than in LP samples. This is consistent with the higher number of lower expressed miRNAs in ER-negative than in ER-positive tumor samples observed by others [24], [49].

As differential expression does not imply causality, we carried out functional assays to validate and characterize the effect of individual miRNAs on proliferation. In the cases of miR-130b and miR-19a, up regulated in HP (with positive correlation with the cell-cycle genes), and miR-449a, miR-299, miR-154 and miR-145, downregulated in HP (with negative correlation with the cell-cycle genes), the effect of miRNA over-expression on proliferation was confirmed in cell lines. We therefore propose that for these miRNAs the association with proliferation is not only manifested in the transcriptomics level but rather they are likely to be drivers of the process. The strongest effect was seen for miR-449a. In prostate cancer cell lines miR-449a was shown to have growth suppressing activity partly through inhibition of HDAC-1 expression [50]. The expression patterns of miR-449a and of HDAC-1, in our cohort, are anti-correlated (Pearson's r = −0.26) and it might be that the mechanism is similar in breast cancer. It is therefore of interest to characterize its relationship to proliferation and assess its therapeutic potential. Consistent with our findings, this recent study [50] also reports miR-145 to inhibit proliferation. We note that Blenkiron et al. [24] observed higher expression of miR-145 in luminal-A samples.

There is an apparent disagreement between the functional proliferation assay results for several other miRNAs (e.g. miR-18a/b). Since association does not imply causality this apparent disagreement is, in fact, expected. In addition, this disagreement can be a result of the complexity of transferring observations from individual cell-lines to clinical tumor cohorts. For the case of miR-18a/b we note that [27] showed that this miRNA and the miRNA cluster it resides in (miR-17-92 cluster) are activated by E2F. Therefore, it is reasonable to expect its expression level to be driven by the cell-cycle process rather than to be a determinant of that process.

Analyzing miRNA expression with respect to extracellular matrix component signature of the studied cohort, we found miR-29c to be the most prominently differentially expressed. It is under-expressed in the ECM1 class and over-expressed in the luminal-A subtype. Predicted targets of the miR-29 family are enriched with genes associated with cell-adhesion and show significant anti-correlation to the expression of miR-29c which points to a direct involvement of miR-29c in regulating cell-adhesion. Over-expression of the miR-29 family was shown to revert aberrant methylation patterns in lung cancer [51], and recently it was shown that miR-29 can induce apoptosis in a TP53 dependent manner [52]. In our cohort we also found miR-29c to be significantly under-expressed in proliferative samples (Table S5), which may suggest breast tumor suppressive activity mediated by the regulation of the ECM related genes.

We have run univariate Cox analysis to assess the association of miRNAs and survival in the entire cohort. We have not found any significant association of any of the tested miRNAs, after correcting for the multiple testing. We do find miR-150 to be associated to survival in part of the cohort as described above.

### Conclusion

We introduce a dataset of mRNA and miRNA expression profiles measured in a well studied patient cohort. We show that miRNAs can distinctly differentiate between tumor subtypes and various clinical sub-classifications. In addition, we present experimental support linking some miRNAs to proliferation. Finally, we show that miRNAs can act as reliable proxies to the activity of known biological processes related to breast cancer progression such as cell-cycle, immune response and cell adhesion.

## Materials and Methods

### Patient characteristics and classifications

The 101 breast cancer patients in this study are part of a cohort previously described [53]. The study was approved by the Norwegian Regional committee for medical research ethics, Health region II (reference number S-97103). All patients have given written consent for the use of material to research purposes. Total RNA isolation was performed using TRIZOL (Invitrogen) as described previously [54]. The mRNA expression derived subtype classification has previously been performed and presented [8]. The same is true for the extracellular matrix (ECM) based classification [29].

### Expression profiling

#### miRNA microarray hybridization

miRNA profiling from total RNA was performed using Agilent Technologies “Human miRNA Microarray Kit (V2)” according to manufacturer's protocol. Scanning on Agilent Scanner G2565A and Feature Extraction (FE) v9.5 was used to extract signals. Excluding two samples, experiments were performed using duplicate hybridizations (99 samples) on different arrays and time points. miRNA signal intensities for replicate samples were averaged and log2 transformed. The expression levels were normalized to the 75^th^ percentile. That is, the expression levels in each sample, *i*, were multiplied by a constant, *c_i_*, such that the 75^th^ percentile of the expression levels in that sample will equal to a constant *c* – the 75^th^ percentile in the entire dataset. miRNA expression status was scored as present or absent for each gene in each sample by default settings in FE v9.5. miRNAs in samples that were run in replicates were considered present if scored in one of the two arrays. The microarray contains probes for 76 viral and 723 human miRNAs (based on miRBASE v10.1). We filtered out all miRNAs that were detected in less than 10% of the samples. This filtering resulted in 489 miRNAs considered to be expressed in this set of human breast tumors and used in further analysis steps. For these miRNAs, all expression values were used for further analysis. Thus, no missing values were used. The miRNA expression data is MIAME compliant and have been submitted to the Gene Expression Omnibus (GEO) with accession number GSE19536.

#### miRNA expression using RT-PCR

Quantification of nine selected mature miRNAs was performed on 20 samples with TaqMan® MicroRNA Assays (Applied Biosystems). The miRNAs selected for this validation were miR-17-5p, miR-18a, miR-18b, miR-19a, miR-29c, miR-34c-5p, miR-142-3p, miR-150 and miR-449a, and the endogenous control used was RNU6B. RT-PCR reactions were carried out using the manufacturer's recommendation. In brief, 10 ng of total RNA was reverse transcribed using the TaqMan® MicroRNA Reverse Transcription kit (Applied Biosystems) with miRNA specific RT-primers (Applied Biosystems). Quantitative Real-Time PCR was performed following the manufacturer's recommendation in triplicates on a 7900 HT Fast Real-Time PCR System (Applied Biosystems) with a standard absolute quantification thermal cycling program and using the SDS 2.3 software (Applied Biosystems) to determine the cycle threshold (C_t_). The endogenous control was used for normalization. Pearson correlation was used to investigate the correlation between the microarray and RT-PCR quantification approaches (Figure S6).

#### mRNA microarray hybridization

mRNA profiling from total RNA on the same (TRIZOL extracted) samples were performed on an Agilent catalogue design whole human genome 4x44K one color oligo array. Scanning was performed on Agilent Scanner G2565A and signals were extracted using Feature Extraction v9.5. Data were log2 transformed, non-uniform spots were excluded. Population outliers were excluded when averaging replicated probes. Probes that are missing on more than 10 arrays were excluded. Quantile normalization was performed in R using normalizeBetweenArrays from the LIMMA library [55] and missing values imputed using LLS imputation (R: LLSimpute from the pcaMethod library with k = 20) [56]. The mRNA expression data is MIAME compliant and have been submitted to the Gene Expression Omnibus (GEO) with accession number GSE19783.

### Statistical analyses

#### Hierarchical clustering

Hierarchical clustering was carried out using MeV data analysis tool (ver. 4.4, http://www.tm4.org/). The 100 miRNAs with highest expression variance were selected. Average linkage clustering was carried out on both samples and miRNAs, using Spearman correlation as a distance measure. miRNA expression values were normalized for visualization purposes.

#### Differential expression

The assessment of miRNA differential expression when considering the comparison of any two classes was performed using TNoM (Threshold Number of Misclassifications) [57], [58]. Briefly, TNoM counts the number of misclassified samples, according to the given gene expression pattern, and uses combinatorics to compute the exact a-parametric p-value. To assess which genes are significant we used the FDR method [25] based on the exact p-value calculations for TNoM.

#### Enrichment analysis

To assess the enrichment of a fixed gene set within a ranked list of genes we use the *minimum hypergeometric* (mHG) statistics [59]. Briefly, consider a ranked list of genes: g_1_,…,*g_N_* and a given subset of genes *H*. We define a label vector *λ*  =  *λ*_1_,…,*λ_N_* ∈ [0, 1]*^N^* according to whether the ranked genes belong to *H*. Namely, *λ_i_*  =  1 iff *g_i_* ∈ *H,* for every *i*. The mHG score is then defined as:
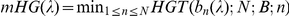



Where
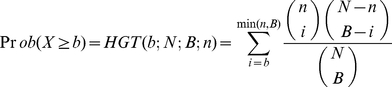
is the tail of the. hypergeometric distribution for a random variable X, and
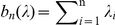
mHG p-values presented herein are exact and do not require correction for multiple thresholds tested [59].

In this paper we use GO terms [60], MSigDB sets [61] and miRNA target sets [30] to define different sets of genes (playing the role of H above). GO enrichment analysis was performed using the GOrilla web tool [62], which takes as input a ranked list of genes.

The following ranking schemes are used in the analyses performed in this study (see Figure S7): (A) Differential expression – using TNoM or other scores, as described above. (B) Correlation to pivot miRNA – for a specific miRNA, called the pivot miRNA, mRNAs were ranked according to the correlation of their expression pattern across the entire cohort (101 samples) to the pivot miRNA expression pattern. To obtain the results presented here we used Spearman's correlation. (C) miRNA target prediction – for a specific miRNA, genes were ranked according to how likely they are to be targeted by the miRNA. Context score values, taken from TargetScan V5.1 target prediction tool [30], were used as prediction scores.

In the case of miRNA target enrichment, the genes were ranked according to their anti-correlation to a pivot miRNA. The top 2000 targets of the pivot miRNA were then tested for enrichment amongst the top anti-correlatees. Enrichment in this case indicates a significant anti-correlation between the expressions of the miRNA and the expression of its targets.

#### Survival analysis

The univariate Cox scores for each of the 489 miRNAs was calculated using SAS. The log-rank calculation for miR-150 was performed using Matlab version 2008b (Mathworks, Inc).

### Assessment of proliferation

Formalin-fixed paraffin-embedded tissue was available from 93 of the 101 patients as part of a Tissue Micro Array (TMA). Briefly, the TMA was composed of three 0.6 mm cores from each of the tumor specimen assembled in a recipient paraffin block using a manual device from Beecher Instruments, Silver Spring, USA. Immunohistochemistry (IHC) was performed with antibody directed towards Ki67 (MIB1 from DAKO, diluted 1∶100) using the Envision+ detection system (DAKO). The slides were scored visually using a conventional microscope and grouped by level of positivity into three groups; negative (≤1% stained cells), moderate (1-10%) and high (>10%) regardless of intensity. Samples with no interpretable cores were excluded (three patients). The highest value was used if the three cores from a patient showed discrepant scores. In total, 90 patients had interpretable Ki67 IHC staining; 29 were classified as negative, 39 classified as moderate and 22 classified as high Ki67 (summary of scoring results presented in Table S10) [53].

Whole tissue sections were available from 92 patients, and the mitotic count was assessed by microscopy as part of estimating the histological grade. Briefly, the number of mitoses in 10 high power fields were counted and the samples were classified as having low (0–5 mitoses), moderate (6–11 mitoses) or high (>11 mitoses) mitotic index (MI). Out of 95 samples, 39 had low MI, 21 had moderate MI and 32 had high MI.

Proliferation groups were created by dividing the samples in two groups. Samples with a high score on both Ki67 and mitotic index or high and moderate were considered highly proliferative (24 samples). Samples that scored low/negative on both Ki67 and mitotic index or low and moderate were considered weakly proliferative samples (35 samples) (Table S10).

### Pre-miR transfections and scoring

MCF-7 cells were obtained from Interlab Cell Line Collection (ICLC, Genova, Italy) and BT-474 from American Type Culture Collection (ATCC, Manassas, VA, USA). For lysate microarray (LMA) screening, the MCF-7 and BT-474 cells were transfected with 20 nM human Pre-miR™ miRNA Precursor library v2 (Ambion Inc., Austin, TX) as previously described [28]. Thereafter, the cells were lysed and printed on nitrocellulose-coated microarray FAST™ slides (Whatman Inc., Florham Park, NJ). Ki67 was detected by staining the slides with Ki67 antibody (#M7240, Dako, Glostrup, Denmark) followed by exposure to Alexa Fluor 680 -tagged secondary antibody (Invitrogen Inc., Carlsbad, CA). For total protein measurement, the arrays were stained with Sypro Ruby Blot solution (Invitrogen Inc.). The slides were scanned with Tecan LS400 (Tecan Inc., Durham, NC) microarray scanner and Odyssey Licor IR-scanner (LI-COR Biosciences, Lincoln, NE) to detect the Sypro and Ki67 signals, respectively. Array-Pro Analyzer microarray analysis software (Median Cybernetics Inc., Bethesda, MD) was used for analyzing the data. For each miRNA the signal intensity was normalized to a negative control miRNA (z-score). miRNAs that gave at least one hit with a z-score >2 or <-2 as well as both z-scores >1 or <-1 at both 48 h and 72 h were considered as having an effect on proliferation.

## Supporting Information

Figure S1 Expression profiles of miR-9*, miR-29c and miR-190b in subtypes. Expression profiles (signal intensities) of miR-9*, miR-29c and miR-190b ordered by subtypes. Note that Y-axes in different panels are in different scales.
(EPS)

Figure S2 **Proliferative miRNAs versus cell-cycle related enrichments.** The plot shows the scores for each miRNA, where each miRNA is represented by a dot. On the Y-axis the differential expression score is –log(p-value) if the miRNA is upregulated and log(p-value) if the miRNA is down regulated, yielding positive and negative scores according to differential expression between the high and low proliferative groups. Significance of differential expression is calculated using TNoM as described in Materials and Methods. (A) “Positive regulation of mitotic cell cycle” score is –log(p-value) if CC genes are enriched in the miRNA positively correlated genes, and log(p-value) if CC genes are enriched in the miRNA negatively correlated genes. P-value for CC enrichment is calculated using the mHG statistic as described in Materials and Methods. Here CC genes are those annotated in GO as “positively regulation of mitotic cell cycle”. (B) “Negative regulation of S phase of mitotic cell cycle” scores are calculated in the same manner as “Positive regulation of mitotic cell cycle” scores. The difference is in the definition of CC genes. Here we use genes annotated in GO as “Negative regulation of S phase of mitotic cell cycle”.
(EPS)

Figure S3 **Association of miR-29c with extracellular matrix.** (A) Extracellular matrix miRNA differential expression. miRNAs ordered by significance of differential expression between two reciprocal extracellular matrix classes ECM1 and ECM4. For visualization expression values of each miRNA were linearly stretched. miR-29c shows the highest significance of differential expression between the two classes (TNoM p<4E-5, see Table S8 for full list). (B) miR-29c is anti-correlated to its mRNA targets. All mRNAs were ranked according to their anti-correlation to miR-29c expression profile. The absolute signal intensities of miR-29c are presented in the top bar. The top 50 anti-correlated genes are ordered from top to bottom. We find a significant enrichment of miR-29c targets, as derived from TargetScan V5.1, in the anti-correlated gene ranking (mHG p<3E-11). The color bar beneath the heatmap illustrates the different subtypes. (C) GO enrichment in miR-29c anti-correlated genes. The figure depicts the GO enrichment result, as carried out by GOrilla [62]. We observed an enrichment of terms related to extracellular matrix (e.g. cell-adhesion).
(EPS)

Figure S4 **GO enrichment in miR-29c targets.** Target prediction context scores of miR-29c were taken from TargetScan V5.1. We find enrichment of several GO terms in the high scoring genes, with respect to miR-29c targets, using GOrilla web tool [62]. The graph is color coded according to degree of enrichment.
(EPS)

Figure S5 **miR-150 and survival.** ER-/non-ERBB2 enriched patients from our cohort were divided to two groups: patients with high expression of miR-150 (above the average in the entire cohort which was 1041) and patients with low expression of miR-150 (below average). Using a log-rank test we found that high expression of miR-150 is predictive of better prognosis (log-rank p<0.085) in the ER-/non-ERBB2 enriched patients.
(EPS)

Figure S6 **RT-PCR analysis of miRNA expression.** Each panel displays Agilent expression (vertical axis) versus negative TaqMan expression (horizontal axis) for a miRNA on all 20 samples. Two lines are shown in each panel: the least squares fit to the data (green) and a robust regression line found by iteratively reweighted least squares with a bisquare weighting function (magenta). Pearson's correlation coefficients and corresponding p-values are shown above each panel.
(EPS)

Figure S7 **Workflow.** Schematic overview of the data analysis methods applied. (A) Differential expression – using TNoM or other scores. (B) Correlation to pivot miRNA – for a specific miRNA, called the pivot miRNA, mRNAs were ranked according to the correlation of their expression pattern across the entire cohort (101 samples) to the pivot miRNA expression pattern. (C) miRNA target prediction – for a specific miRNA, genes were ranked according to how likely they are to be targeted by the miRNA. A, B and C all lead to ranked lists of genes which are analyzed using the mHG method.
(EPS)

Table S1 **Characterization of the samples.**
(XLS)

Table S2 **miRNAs differentially expressed between basal-like and luminal-A like samples.**
(XLS)

Table S3 **miRNAs differentially expressed between TP53 WT and mutant and between ER-positive and ER-negative.**
(XLS)

Table S4 **Enrichment levels of miRNA-GO associations.**
(XLS)

Table S5 **miRNAs significantly associated with proliferation.**
(XLS)

Table S6 **Proliferation associated miRNAs in ER-positive and ER-negative samples.**
(XLS)

Table S7 **LMA screen of Ki67.**
(XLS)

Table S8 **miRNAs differentially expressed between ECM1 and ECM4.**
(XLS)

Table S9 **miRNAs associated with survival.**
(XLS)

Table S10 **Proliferation scoring scheme.**
(XLS)
