# Correction: miRNA-mRNA Integrated Analysis Reveals Roles for miRNAs in Primary Breast Tumors

**DOI:** 10.1371/annotation/a20454a7-ddc4-4657-b73c-185bd6ec6977

**Published:** 2013-09-10

**Authors:** Espen Enerly, Israel Steinfeld, Kristine Kleivi, Suvi-Katri Leivonen, Miriam R. Aure, Hege G. Russnes, Jo Anders Rønneberg, Hilde Johnsen, Roy Navon, Einar Rødland, Rami Mäkelä, Bjørn Naume, Merja Perälä, Olli Kallioniemi, Vessela N. Kristensen, Zohar Yakhini, Anne-Lise Børresen-Dale

Supporting Information Figures S6 and S7 were incorrectly switched. The image currently appearing as Figure S6 corresponds to the title and legend for Figure S7, and the image currently appearing as Figure S7 corresponds to the title and legend for Figure S6. The titles and legends themselves are in correct order. 

